# Coupling of emergent octahedral rotations to polarization in (K,Na)NbO_3_ ferroelectrics

**DOI:** 10.1038/s41598-017-15937-x

**Published:** 2017-11-15

**Authors:** I. Levin, V. Krayzman, G. Cibin, M. G. Tucker, M. Eremenko, K. Chapman, R. L. Paul

**Affiliations:** 1 National Institute of Standards and Technology, Gaithersburg, MD 20899 USA; 2 Diamond Light Source, Didcot, OX11 0DE UK; 3 Spallation Neutron Source, Oak Ridge National Laboratory, Oak Ridge, TN 37830 USA; 4 Advanced Photon Source, Argonne National Laboratory, Lemont IL, 60439 USA

## Abstract

Perovskite potassium sodium niobates, K_1−x_Na_x_NbO_3_, are promising lead-free piezoelectrics. Their dielectric and piezoelectric characteristics peak near *x* = 0.5, but the reasons for such property enhancement remain unclear. We addressed this uncertainty by analyzing changes in the local and average structures across the *x* = 0.5 composition, which have been determined using simultaneous Reverse Monte Carlo fitting of neutron and X-ray total-scattering data, potassium EXAFS, and diffuse-scattering patterns in electron diffraction. Within the A-sites, Na cations are found to be strongly off-centered along the polar axis as a result of oversized cube-octahedral cages determined by the larger K ions. These Na displacements promote off-centering of the neighboring Nb ions, so that the Curie temperature and spontaneous polarization remain largely unchanged with increasing *x*, despite the shrinking octahedral volumes. The enhancement of the properties near *x* = 0.5 is attributed to an abrupt increase in the magnitude and probability of the short-range ordered octahedral rotations, which resembles the pre-transition behavior. These rotations reduce the bond tension around Na and effectively soften the short Na-O bond along the polar axis – an effect that is proposed to facilitate reorientation of the polarization as external electric field is applied.

## Introduction

Perovskite-like potassium sodium niobates, K_1−x_Na_x_NbO_3_ (KNN), are on the short list of commercially viable lead-free piezoelectrics^1,2^. The technologically-relevant K-rich part of the KNN phase diagram^3,4^ is dominated by polymorphic phase transitions that originate in KNbO_3_. This end compound on cooling undergoes a sequence of changes from the high-temperature paraelectric cubic (**C**) phase to the lower-temperature ferroelectric tetragonal (**T**), orthorhombic (**O**), and rhombohedral (**R**) polymorphs; at room temperature, the **O** phase is stable. In the average structures, these transitions are manifested by cooperative polar displacements of the cations along the 〈001〉 (**T** phase), 〈110〉 (**O**), and 〈111〉 (**R**) directions of the cubic phase.

The temperatures of the **C**↔**T** and **T**↔**O** transitions remain flat across nearly the entire compositional range. Substitution of K (ionic radius 1.64 Å^5^) by smaller Na (1.39 Å) eventually induces additional phase changes associated with octahedral rotations, which occur for *x* ≥ 0.6. The K- and Na-rich parts of the diagram are thought to be divided by a morphotropic phase boundary (MPB) at *x* ≈ 0.5, which separates the ferroelectric K-rich **O** and Na-rich monoclinic **M** phase fields. The piezoelectric and dielectric properties peak at this boundary^6–8^ and, therefore, most studies dealing with the development of practical KNN ceramics have focused on the *x* ≈ 0.5 composition. Even more significant enhancement of properties can be achieved by doping KNN with other species to shift the **T**-**O** transition down to room temperature^2^. According to the published phase diagram^2–4^, the untilted **M** structure is stable over a narrow compositional range between *x* ≈ 0.5 and *x* ≈ 0.6, while for higher Na content it transforms to another monoclinic phase, which combines ferroelectric displacements with in-phase octahedral rotations about the pseudo-cubic axis perpendicular to the polarization direction.

Despite the vast literature on KNN, the nature of the proposed MPB at *x* ≈ 0.5 remains ill understood. Room-temperature power diffraction patterns for all the compositions with *x* ≤ 0.6 can be indexed using a primitive monoclinic unit cell (*a*_*m*_ ≈ *b*_*m*_ ≈ *c*_*m*_ ≈ a_c_ ≈ 4 Å, β ≈ 90°; here subscripts “*m”* and “*c”* refer to the monoclinic and cubic cells, respectively)^9–11^. Some studies reported a discontinuous change of the lattice parameters near *x* = 0.5^3,9^, whereas others^7,10^ observed a continuous trend. If *a*_m_ = *c*_m_, this primitive monoclinic cell becomes equivalent to a reduced version of the *A*-centered orthorhombic (subscript “*o*”) cell with lattice parameters *a*_o_ = *a*_c_, *b*_o_ = *a*_c_√2, *c*_o_ = *a*_c_√2, which is commonly used to describe the **O** structure. Therefore, the width of the *h*_m_00/00 *l*_m_ diffraction peak, which depends on the difference between *a*_m_ and *c*_m_, in principle, can serve as an indicator of the monoclinic distortion.

Ahtee & Glazer^11^ and Baker *et al*.^4^ claimed an onset of a small (<0.2%) *a*_m_-*c*_m_ splitting at *x* = 0.5, ascribing this effect to the presence of an **O**↔**M** MPB^4^. This monoclinic distortion (**M**_C_^12^,) has been attributed to deviation of the polar displacements from the mirror symmetry plane normal to the *b*-axis of the **O** *Amm2* structure. The birefringence measurements on single crystals of KNN^13^ suggested a different monoclinic structure (**M**_A_/**M**_B_) for *x* = 0.5, in which the polarization vector deviates from the mirror plane normal to the *a*-axis in the *Amm2* setting. None of these monoclinic models have been validated by structural refinements, presumably because of the vanishingly small, if real, distortions from the orthorhombic cell.

The relationship between the proposed MPB and the properties is also not intuitive. Typically, an MPB that leads to an enhanced piezoelectric response separates two ferroelectric structures with strongly distinct orientations of polarization, as it occurs, for example, in tetragonal and rhombohedral phases, respectively^14^. While the exact mechanism of this enhancement may still be debatable, a thermodynamic analysis suggests that the directional polar anisotropy vanishes at such a boundary, enabling easy rotation of a polarization vector under applied electric field^15^. In KNN, the structural differences across the proposed MBP appear to be too subtle for such a mechanism to be significant. Adding to this controversy, the detailed character of the compositional dependence of KNN’s properties varies among different studies from a sharp, spike-like peak, consistent with a phase boundary effect, to a broader maximum that could simply reflect the presence of several competing phenomena.

In the present study, we determined the room-temperature structures of KNN (*x* < 0.6) using a Reverse Monte Carlo (RMC) method^16,17^ to simultaneously fit several types of experimental data that are sensitive to both local and average atomic displacements. The results demonstrated that the local Nb displacements in the **O** structure occur approximately along the average polar axis, in contrast to the established order-disorder model of phase transitions in KNbO_3_. The Na cations, which reside in the oversized cube-octahedral cages required to accommodate the larger K ions, are also offset along the polar axis and their displacements amplify the shifts of the neighboring Nb. Near *x* = 0.5, an abrupt increase in the magnitude of short-range ordered octahedral rotations reduces the off-centering of Na along the polar direction and promotes disorder of the Na displacements – an effect that is proposed to be at the origin of the enhanced dielectric and piezoelectric properties.

## Results and Discussion

We analyzed ceramic samples of KNN with *x* = 0, 0.47, 0.53, and 0.58 prepared using conventional solid-state synthesis (See supplementary materials for details of synthesis and characterization.) The local structure in these samples has been determined using the development version of the RMCProfile software^18–22^ to simultaneously fit real, i.e. a pair distribution function (PDF), and reciprocal-space representations of the neutron and X-ray total-scattering data, potassium extended X-ray absorption fine structure (EXAFS), and patterns of diffuse scattering in electron diffraction. The structures were represented with atomic configurations containing 70,000 atoms with random distributions of the Na and K atoms over the cube-octahedral A-sites; three to six configurations have been refined for each composition.

**Figure 1:**
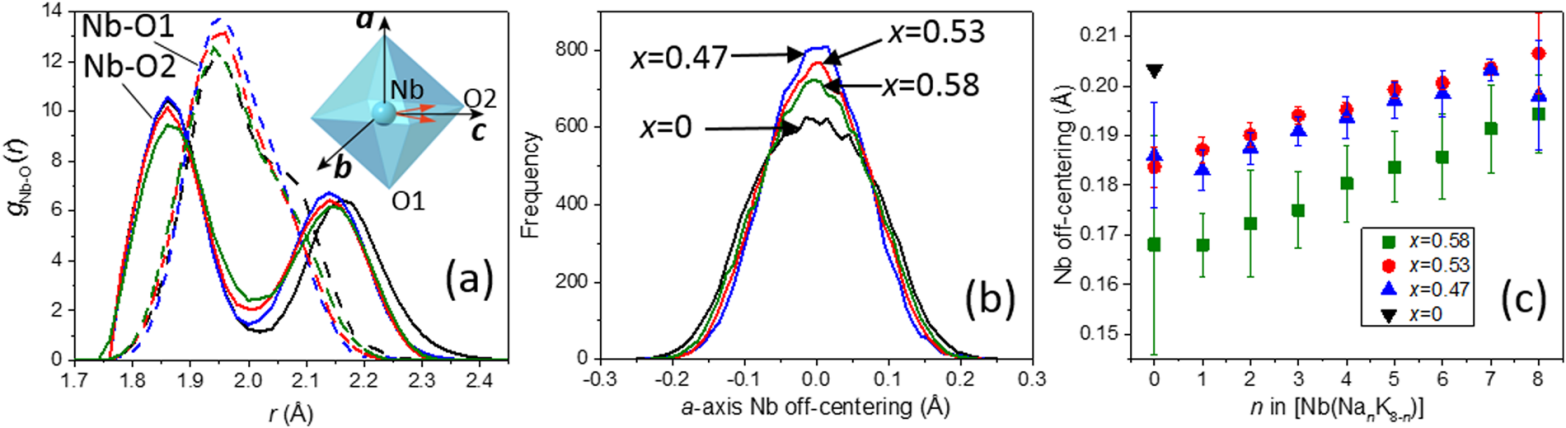
(**a**) Distributions of the Nb-O1 and Nb-O2 bond lengths for the refined configuration of KNbO_3_. The inset shows a schematic rendering of the [NbO_6_] octahedra with the locations of the O1 and O2 atoms and the orthorhombic crystal axes indicated. The Nb displacement is indicated using a red arrow. (**b**) Distributions of the Nb off-centering along the *a*-axis. (**c**) The dependence of the local Nb-off-centering on the number (n) of the Na atoms in the [NbNa_n_K_8−n_] clusters. The errors bars represent a single standard deviation, which corresponds to a distribution of the off-centering for a given n-type cluster.

The lattice parameters reveal no anomalies at *x* = 0.5 and the unit-cell volume shrinks continuously with increasing *x*. Likewise, no systematic trends with composition have been observed for the widths of 200 _m_/002 _m_ diffraction peaks, which were the narrowest for *x* = 0.53. Rietveld refinements of the KNN structures using the neutron diffraction data, which have been performed for both the *Amm2* and *Pm* models, returned similarly satisfactory agreement factors. The atomic displacement parameters (ADPs) for the A-cation and oxygen sites increased monotonically with increasing *x*.

All the KNN samples contained twin-domain variants with domain walls residing predominantly on {100} cubic planes, as expected for the **T**→**O** transition (Supplementary Materials, Fig. S1); however, these domain structures were somewhat irregular, especially for *x* = 0.53, and occasional {110} domain walls also were observed. Such a coexistence of the {100} and {110} wall orientations would support the monoclinic symmetry because the **T**→**M** transition permits both types of domain walls, whereas only the {100} interfaces are allowed in the **O** phase^23^. However, only the occasional presence of the {110} domain walls and the overall irregularity of the domain structures precludes their conclusive interpretation in terms symmetry.

Electron diffraction patterns from the single twin variants revealed diffuse streaks that represented traces of the sheets, which extend perpendicular to the *a*-axis of the *Amm2* structure (Fig. S2). In KNbO_3,_ this diffuse scattering has been attributed to correlated Nb displacements along the octahedral chains parallel to the *a*-axis^24,25^. The same type of correlation displacements appears to exist throughout KNN. The diffraction patterns for *x* = 0.58 featured additional weak *0kl* spots with odd *k* and *l* indices, which must be absent in the untilted **O** or **M** structures. We have attributed these spots to the *in-phase* octahedral rotations about the *a*-axis. Evidently, the *x* = 0.58 composition resides close to the tilting phase-transition boundary^4^ and, therefore, the superlattice reflections are too weak and diffuse to be detectable by X-ray or neutron powder diffraction.

The total scattering and EXAFS data exhibit only small albeit systematic changes as *x* increases, which are manifested primarily in broadening of the PDF and EXAFS peaks (Fig. S3, Fig. S4). Overall, all the data confirm that the structural differences among the KNN compositions are subtle. An example of the agreement between the experimental and calculated signals produced by simultaneous fitting of all the datasets in RMCProfile is shown in Fig. S5. The compositional dependence was closely reproduced.

The Nb-O (Fig. 1a) and Nb-Nb (not shown) distance distributions remain nearly unchanged for the KNN compositions. For all the x-values, the Nb cations are displaced predominantly along the polar *c*-axis of the orthorhombic unit cell (Fig. 1a), as in the average structure. (Note: The total Nb-O doublet in the *G*_N_(*r*) can be fitted satisfactorily using two Gaussian peaks with approximately equal areas which could incorrectly imply that the Nb atoms are displaced along the 〈111〉 directions; that is, the inferences made from simple PDF peak fitting can be grossly misleading.) The nearly 5-fold difference between the magnitudes of the *c*-axis (≈0.29 Å) and *a*-axis (±0.06 Å) components of these shifts agrees with the results of the previously reported polarization-dependent EXAFS measurements on single crystals of KNbO_3_^26^. The *a*-axis components of the Nb displacements exhibit strong positive correlations along the [100] octahedral chains, which yield the (100) diffuse scattering sheets (Fig. S3). Large positive correlations also exist among the displacements of other species (i.e., Nb-O, K-O, K-K, Na-O). Overall, the instantaneous displacements in the refined configurations appear to be dominated by the acoustic phonon modes.

The above results provide further insight into the order-disorder model of phase transitions in KNbO_3_, which is based on the interpretation of 〈100〉 diffuse-scattering sheets^24,25,27^. Per this model, Nb cations are locally off-centered along the 8 equivalent 〈111〉 directions even in the **C** phase, so that Nb adopts an 8-site probability density distribution; because of disorder, the net polarization is zero. Then, below the Curie temperature, the local Nb displacements undergo partial ordering with only 4 out of 8 displaced sites occupied in the **T** phase and the structure, on average, becomes polar. Additional ordering to a 2-site distribution is presumed to occur upon the transition to the **O** polymorph, while in the lowest-temperature **R** phase, a completely ordered state is attained with all the Nb cations shifted along a single 〈111〉 direction. Here, we demonstrate that in the **O** phase the local Nb displacements deviate only weakly from the average 〈110〉 polar axis. The distributions of the *a*-axis displacement components is non-Gaussian (Fig. 1b) and for all the KNN compositions the data can be fitted better using two peaks rather than a single peak; however, the peak separation (<0.05 Å) is too small to reliably support a bimodal distribution that is implied by the order-disorder model. Thus, the **O**↔**R** transition must be predominantly of the displacive type since even the local Nb shifts must change their orientation from ≈〈110〉 to 〈111〉. A similar conclusion has been previously inferred from vibrational-spectroscopy studies of phase transitions in KNbO_3_^25^ and from polarization-dependent single-crystal EXAFS measurements^26^.

**Figure 2:**
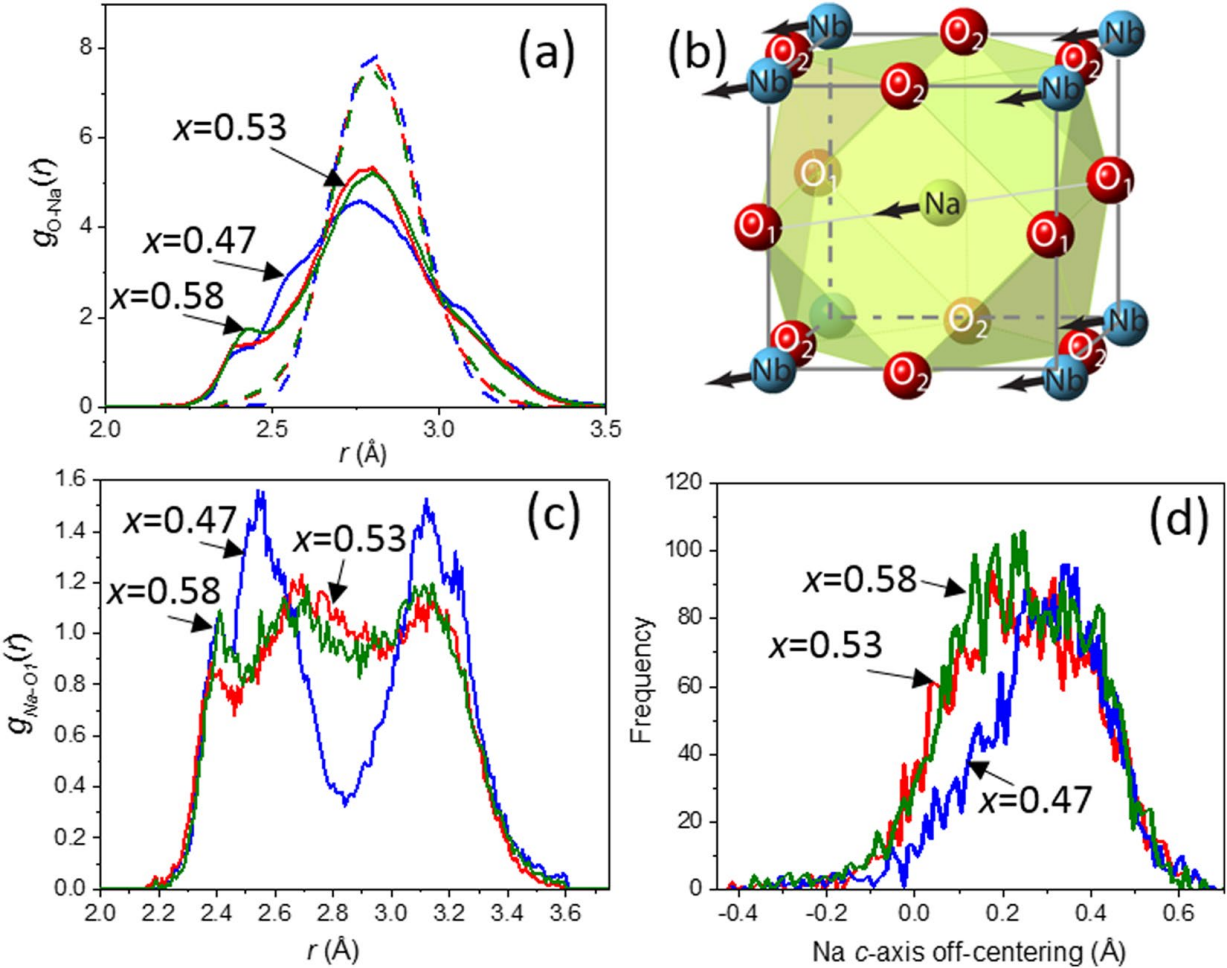
(**a**) Distributions of the Na-O (solid lines) and K-O (dashed lines) bonds lengths for *x* = 0.47 (blue), *x* = 0.53 (red), and *x* = 0.58 (green). (**b**) A schematic rendering of the Na coordination with the Na and Nb displacements indicated using arrows. (**c**) Distributions of the Na-O1 bond lengths. (**d**) Distributions of the Na off-centering along the O1-Na-O1 direction.

As expected from considerations of ionic radii, the coordination environments of the smaller Na cations are considerably more distorted than those of K even while the average Na-O and K-O bond lengths remain similar (Fig. 2a). These distortions, which relieve the tensile Na-O bond strain caused by the oversized [NaO_12_] cages, result from Na displacements along the *c*-axis toward the O1 atoms (Fig. 2b), in accord with the theoretical predictions^28^. The magnitude of the Na off-centering in this direction, which is twice that of K, decreases from ≈0.26 Å for *x* = 0.47 to ≈0.21 Å for both *x* = 0.53 and *x* = 0.58. The resulting bond-valence sum (BVS) values^29^ for Na and K in the KNN samples are ≈0.9 v.u. and 1.8 v.u., respectively; that is, the K-O bonds are strongly compressed. (Note: An ideal BVS value for monovalent K and Na is 1. This BVS value corresponds to strain-free metal-oxygen bonds. Deviations from the ideal BVS indicate either tensile (<1) or compressive (>1) bond strain.) The Na-O distribution changes strongly from *x* = 0.47 to *x* = 0.53, while the differences between *x* = 0.53 and *x* = 0.58 are small (Fig. 2a).

We used the average refined atomic coordinates and Born effective charges adopted from literature^30,31^ to calculate the spontaneous polarization, *P*_s_, for the analyzed compositions (Table 1). The *P*_s_ increases slightly with addition of Na and remains approximately constant for the solid solutions – a trend that is consistent with the flat Curie temperature. The polar Na shifts enhance *P*_s_ relative to KNbO_3_. However, a major factor that contributes to the observed behavior is the Nb off-centering, which remains sustained with increasing *x* despite the shrinking octahedral volume. Analysis of the refined configurations reveals that the local Nb displacements are enhanced for larger fractions of the Na atoms around Nb (Fig. 1c). Apparently, the Na displacements amplify the shifts of the neighboring Nb cations. A similar effect has been observed in (Ba,Ca)TiO_3_ solid solutions in which Ca displacements support the Ti off-centering^22^. Thus, a strong coupling between displacements of small A- and ferroelectric B-site cations appears to be common for perovskite ferroelectrics that combine small and large ions on the A-sites.

**Table 1:** The total spontaneous polarization (C/m^2^) and its components from displacements of the constituent species as calculated from the refined atomic coordinates and Born effective charges^33,34^. The uncertainty, which has been estimated using a series of refined configurations for each composition, is in the third decimal place.

x	Total	Nb	K	Na	O
0	0.38	0.34	0.03		0.013
0.47	0.41	0.36	0.02	0.03	0.001
0.53	0.40	0.35	0.01	0.03	0.002
0.58	0.39	0.34	0.01	0.04	0.003

The distribution of Na-O distances along the *c*-axis changes markedly from *x* = 0.47 to *x* = 0.53. For *x* = 0.47, this distribution exhibits well separated peaks at ≈2.55 Å and ≈3.15 Å, respectively, as expected from the average-structure model, in which Na is offset along the *c*-axis (Fig. 2b,c). In stark contrast, for *x* = 0.53 and *x* = 0.58, the *c*-axis Na-O distributions are represented by several barely resolvable peaks, suggesting significant Na disorder along this direction. The distribution of the magnitude of Na off-centering relative to the O1 atoms positioned along the polar *c*-axis broadens considerably and its center shifts to smaller values as *x* increases from 0.47 to 0.53 (Fig. 2d); for *x* = 0.53 and *x* = 0.58 the distributions are similar. The concurrent broadening of distributions of Na-O distances along the y-axis further supports an abrupt increase in the Na disorder within the *bc* plane.

**Figure 3:**
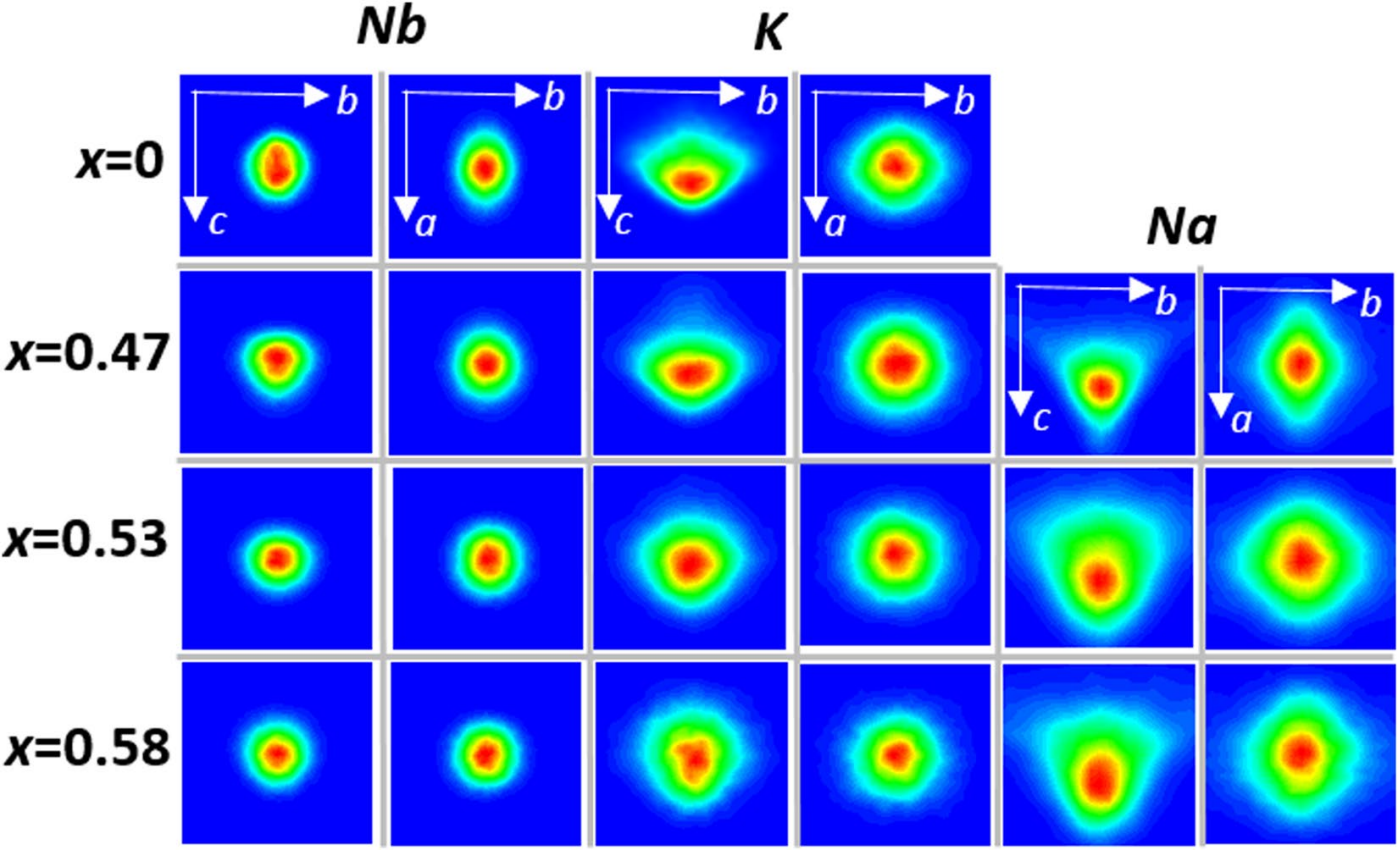
Probability density distributions (PDD) for Nb, K and Na projected onto the *bc* and *ba* planes as a function of *x*. The displayed range of displacements for all the PDDs is ±0.35 Å. Note changes in the spread of the Na displacements from *x* = 0.47 to 0.53; concurrent changes in the anisotropy of the K PDDs also can be observed.

The probability density distributions (PDDs) of all the species, calculated from the refined atomic coordinates, exhibit strongly non-Gaussian, anisotropic shapes. The prominent anisotropy of the A-cation PDDs, which acquire an approximate triangular bi-pyramid shape (Fig. 3), can be rationalized by considering bond-valence requirements of the oxygen atoms. The A-cations are displaced preferentially toward the 3 O1 atoms in the *bc* plane and in between the 4 O2 atoms along the *a*-axis (see Fig. 2b for the schematic of the A-site coordination). These displacements tend to compensate for a certain decrease in the BVS values of the O1 atoms, which is caused by the concerted *c*-axis shifts of the Nb cations. Concurrently, the A-cation motion directly toward the O2 atoms is restricted because the BVS requirements of these oxygens are already satisfied by the large Nb displacements. The A-cation displacements toward the fourth O1 atom (i.e. along the[00-1]_O_ direction) are also restricted because the large cooperative A-cation shifts along the [001]_O_ direction already provide this oxygen with a short A-O1 distance. The anisotropy of the A-cation PDDs is mirrored by the anisotropic oxygen displacements. The PDDs for Nb, O, and K change abruptly between *x* = 0.47 and *x* = 0.53 becoming significantly more symmetric and harmonic. For Na, a marked increase in the magnitude of its displacements along the [010]_O_ and[00-1]_O_ is observed, consistent with changes in the Na-O distance distributions (Fig. 2a,c).

**Figure 4:**
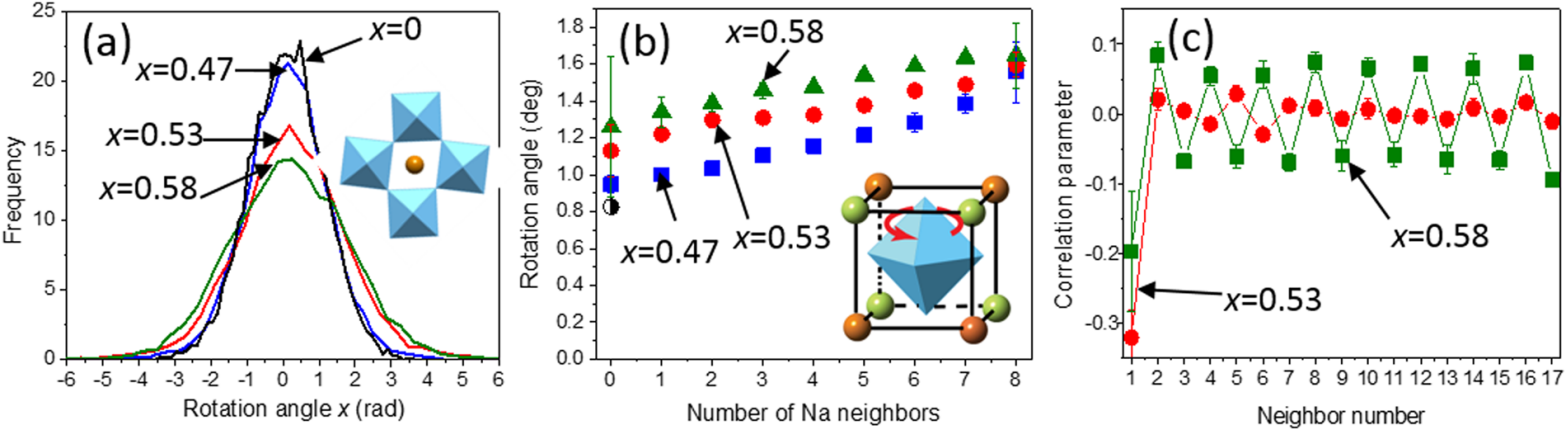
(**a**) Distributions of the magnitudes of the octahedral rotation angles around the *a*-axis. (**b**) The dependence of the absolute value of the octahedral rotation angle (*a*-axis) on the number of Na neighbor atoms, *n*, in the [(NbO_6_)Na_n_K_8−n_] clusters. The errors bars represent a single standard deviation which corresponds to a distribution of the rotation angles for a given n-type cluster. (**c**) Correlation parameters for the *a*-axis rotation angles calculated for octahedral neighbor sequences along the pseudocubic axes in the orthorhombic *bc* plane. The error bars correspond to a single standard deviation estimated from the analyses of several refined configurations for each composition.

The geometric analysis of rotations of the oxygen octahedra performed using the GASP software^32^ reveals abrupt broadening of the distributions of rotation angles between *x* = 0.47 and *x* = 0.53 (Fig. 4a). The contributions of dynamic rotations into the total atomic motion, determined from comparison of two independently refined configurations that represent distinct snapshots of the structure^33^, also increase sharply from ≈10% for *x* ≤ 0.47 to ≈20% for *x* ≥ 0.53. Locally, the octahedral rotation angles increase with the increasing number of the Na atoms, *n*, in the ([NbO_6_]Na_n_K_8−n_) clusters (Fig. 4b). Analysis of the correlations among the rotation angles confirms the presence of long-range order of octahedral rotations about the *a*-axis for the *x* = 0.58 composition (Fig. 4c), in agreement with the presence of the superlattice reflections in the electron diffraction patterns (Fig. S3b); the rotations about two other pseudocubic axes exhibited short-range order only. In contrast, for *x* = 0.53, the ordering of rotations about all three pseudocubic axes is limited to short range (the correlation coefficient for the nearest-neighbor octahedra is ≈0.3); these correlations do not produce any characteristic diffuse scattering that could be identified using electron diffraction, which is consistent with the experimental data (Fig. S3a).

An abrupt increase in the magnitude and probability of octahedral rotations between *x* = 0.47 and *x* = 0.53 can explain the corresponding prominent changes in the Na PDD. In the absence of significant rotations, Na is bound tightly to oxygen atom O1, with which it forms a short bond that stabilizes Na in the oversized A-site cage required by the larger K species. In this case, Na displacement toward O1 is the primary mechanism for relieving the tensile Na-O bond strain because the displacements of other oxygen atoms toward Na are limited by the rigidity of the [NbO_6_] octahedra. This is reflected in the narrow spread of the highly anisotropic Na PDD for *x* = 0.47. The emergent octahedral rotations for *x* = 0.53 provide an additional strain-relief mechanism and therefore weaken the driving force for the Na off-centering along the polar axis while facilitating its shifts in other directions. This is manifested in the increasing fraction of the Na atoms, which exhibit smaller off-centering along the *c*-axis and larger *b*-axis displacements.

We conjecture that the enhanced properties near *x* = 0.5 result from the onset of significant octahedral rotations, which promote directional disorder of the Na displacements that effectively softens the short Na-O bond and reduces the Na off-centering along the polar direction. As Na displacements are intimately coupled to those of Nb (Fig. 1c), this disorder facilitates reorientation of the polarization vector under the applied electric field. The emergence of the large-amplitude short-range ordered octahedral rotations near the tilting phase-transition boundary is akin to the pre-transitional behavior. Possibly, these structural changes are also accompanied by the symmetry reduction from orthorhombic to monoclinic, which would yield the proposed MPB, but this could not be ascertained from the present data.

## Conclusions

In summary, our structural refinements demonstrate intimate coupling between the Na and Nb off-center displacements. The displacements of Na amplify the off-centering of the neighboring Nb. This effect counters the diminishing driving force for the Nb displacements as the octahedral volume shrinks with the increasing fraction of Na; therefore, the Curie temperature and spontaneous polarization remain nearly constant. The displacements of both Na and Nb cations are directed approximately along the polar axis of the average orthorhombic structure, which coincides with the 〈110〉 cubic direction. These results indicate that the existing order-disorder model of phase transitions in KNbO_3_, per which the Nb cations in all four polymorphs (**C**, **T**, **O**, **R**) of this compound are displaced approximately along the 〈111〉 cubic directions, is partly incorrect. For Na concentrations near *x* = 0.5, an increase in the magnitude and probability of octahedral rotations, which still exhibit only short-range order appears to soften the short Na-O bond along the polar axis and promote Na shifts in other directions. This increased disorder of Na displacements, which are coupled to those of Nb, is proposed to ease reorientation of the polarization under an applied electric field, thereby enhancing the dielectric and piezoelectric properties.

## Method

### Sample Synthesis

Ceramic K_1−x_Na_x_NbO_3_ (KNN) samples with *x* = 0, 0.47, 0.53, and 0.58 were prepared using conventional solid-state synthesis starting from K_2_CO_3_ (A.R.), Na_2_CO_3_ (A.R), and Nb_2_O_5_ (99.9985%) as raw materials. Prior to weighing, both K_2_CO_3_ and Na_2_CO_3_ powders were dried in an oven at 180 °C. The raw powders were mixed under acetone using agate mortar and pestle, dried, pressed into pellets, and calcined in air at 850 °C for 8 h. For *x* = 0, this single heating was sufficient to obtain a pure perovskite phase with narrow diffraction peaks. For the solid-solution compositions, the calcined pellets were ground, re-pressed into pellets, and sintered at 1100 °C under a slightly reducing atmosphere, which was previously demonstrated^34^ to minimize Na and K volatility while promoting densification.

### Basic structural and compositional characterization

The samples were initially characterized using X-ray powder diffraction (Cu K_1_ radiation) to confirm phase purity and determine lattice parameters. Given the potential volatility of the alkali metals, chemical compositions of the solid-solution samples were verified using cold neutron prompt gamma-ray activation analysis (PGAA), which has high sensitivity to all the elements in the present system. KNbO_3_ and sodium potassium tartrate, NaKC_4_H_4_O_6_·4H_2_O, were used as standards for measurement of K/Nb and K/Na ratios, respectively. All samples and standards were prepared by sealing 0.5 g to 0.8 g of powder in a bag of FEP Teflon. The measurements were carried out using the NIST cold neutron PGAA spectrometer. The Na peak at 472 keV, K at 770 keV, and Nb at 957 keV were integrated using a commercial peak search program. Counting statistics of 0.5% or better were achieved for all peaks. The measured K/Nb and K/Na ratios agreed within 1% and 2%, respectively, with their nominal values.

### Transmission electron microscopy

Transmission electron microscopy has been performed in a conventional instrument operated at 200 kV. The samples were prepared either by dispersing suspensions of crushed powders on lacey carbon-coated grids or by mechanical polishing of sintered pellets followed by ion thinning (4 kV) at the liquid nitrogen temperature until perforation with a final ion polishing completed at 100 V to remove the damaged surface layers.

### Neutron and X-ray total scattering Measurements and EXAFS

Neutron total scattering data were collected using the Polaris instrument at ISIS (Science and Technology Facilities Council, UK). The data were reduced in the GUDRUN software to obtain the properly normalized neutron (*N*) scattering function, *S*_*N*_(*Q*), and the related pair distribution function (PDF), *G*_*N*_(*r*). K and Na exhibit nearly identical neutron scattering lengths and cannot be differentiated using neutron diffraction. Therefore, we augmented the neutron total scattering data with the K *K*-edge EXAFS (E = 3608 eV), which has been measured at the beamline B18 of the Diamond Light Source (Science and Technology Facility Council, UK). The EXAFS measurements were performed at room temperature in a fluorescence mode and the data were reduced in Athena^35^; preliminary EXAFS fits were accomplished in Artemis with the scattering amplitudes and phases calculated using FEFF8^36^. KNbO_3_ structure was used as a reference for determining the value of *E*_0_. Additionally, X-ray total scattering data were measured to emphasize the Nb-Nb correlations. These measurements were performed at the beamline 11-ID-B of the Advanced Photon Source, Argonne National Laboratory, using an incident beam energy of 60 kV. The data were reduced in the PDFGetX3 software^37^ to extract the *S*_*X*_(*Q*) and *G*_*X*_(*r*).

### Structure Refinements

Rietveld refinements were completed by fitting the neutron diffraction data in GSAS^38^. The resulting average-structure models were used to generate initial atomic configurations for RMC refinements, performed in the development version of the RMCProfile software^19–22^. Despite predictions of a large miscibility gap in KNN^39^, experimentally no indications of significant clustering of Na and K has been observed. Therefore, we assumed a random distribution of these species. Atomic configurations of ≈70,000 atoms were used to simultaneously fit *S*_*N*_(*Q*), *G*_*N*_(*r*), *S*_*X*_(*Q*), *G*_*X*_(*r*), neutron Bragg intensities, K EXAFS, and electron diffuse scattering patterns representing four distinct sections of reciprocal space. The convergence of these fits to within the levels of statistical uncertainties was achieved after each atom has been moved on average ≈25 times. The instrument-resolution effects on both *Q*- and *r*-space data were accounted for during RMC fits as described in^21^. A resolution function was determined by measuring the NIST Si SRM 640c. In RMCProfile, X-ray *G*_X_(r) is obtained as the Fourier transform of the *S*_X_(*Q*), which is calculated from the atomic coordinates with the *Q*-dependence of X-ray scattering cross-sections included.

For electron diffraction, only the geometric locus of diffuse intensity, not the intensity values are fitted, as the accuracy of the latter is compromised by the multiple-scattering effects. The experimental electron diffraction patterns contained parasitic features, which would complicate the fit. Considering a simple geometric shape of the diffuse-intensity distributions, we used simulated patterns as an input instead, as described previously^22^. These simulated patterns were generated to closely reproduce the locus of the diffuse intensity, the widths of the diffuse streaks, and the *Q*-dependence of scattered intensity. Combining several types of data was critical not only for resolving K and Na but also for improving the fidelity of the obtained structural models, given that only subtle differences existed between the atomic arrangements in the analyzed compositions. According to our previous studies using simulated data for which the structure is known^21,40,41^, this approach enables recovery of fine details of 3-D atomic arrangements, including atomic displacements and their correlations, in complex perovskites and their solid solutions.

### Data Availability

The datasets generated during and/or analyzed during the current study are available from the corresponding author on reasonable request.

## Electronic supplementary material

Supplemental Information
