# Supplementary material for: Coupling of emergent octahedral rotations to polarization in (K,Na)NbO3 ferroelectrics
Source: Sci Rep. 2017 Nov 15;7:15620. doi: 10.1038/s41598-017-15937-x (PMC5688101; doi:10.1038/s41598-017-15937-x)
Supplement: Supplementary file 1 — Supplemental Information [file 41598_2017_15937_MOESM1_ESM.pdf]

Supplementary Information

(I. Levin, V. Krayzman, G. Cibin, M. G. Tucker, M. Eremenko, K. Chapman, R. L. Paul, Coupling of emergent octahedral rotations to polarization in $(K,Na)NbO_3$ ferroelectrics)

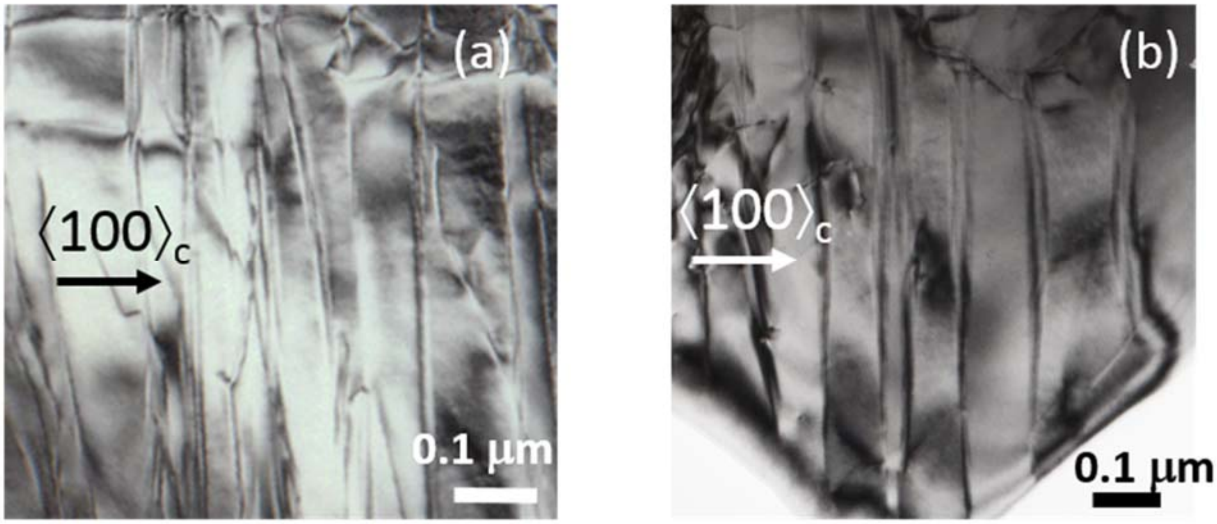

Fig. S1: Diffraction-contrast TEM images of the domain structures in (a) x=0.53 and (b) x=0.58. In both cases, the domain walls reside predominantly on {100} pseudocubic planes.

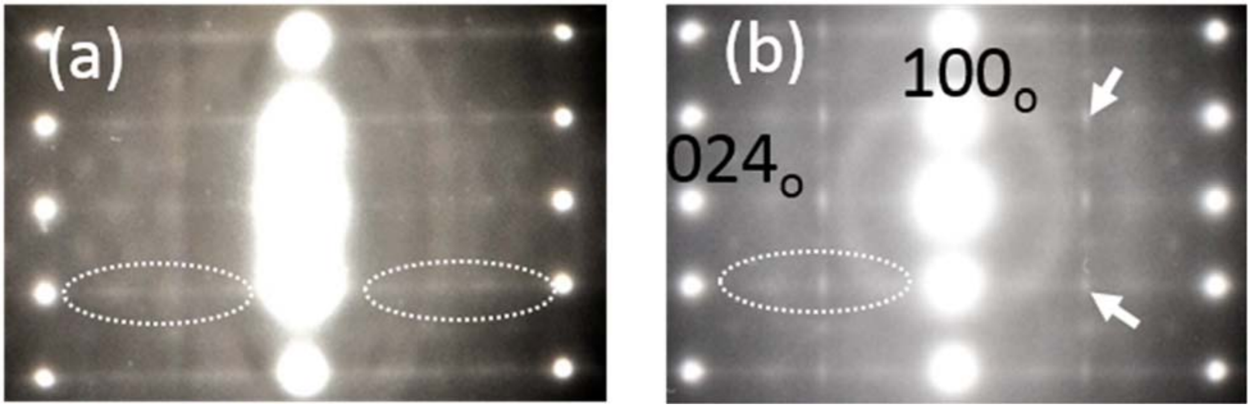

Fig. S2: Selected-area electron diffraction patterns recorded from single twin domains with (a) x=0.53 and (b) x=0.58 in $[02\text{-}1]_o$ zone-axis orientation. The diffuse streaks are encircled with dotted lines. The superlattice reflections at k=$[012]_o$* (asterisk indicated reciprocal space) attributed to the in-phase octahedral tilting are marked using arrows. These reflections equivalent to ½hh0 in cubic index.

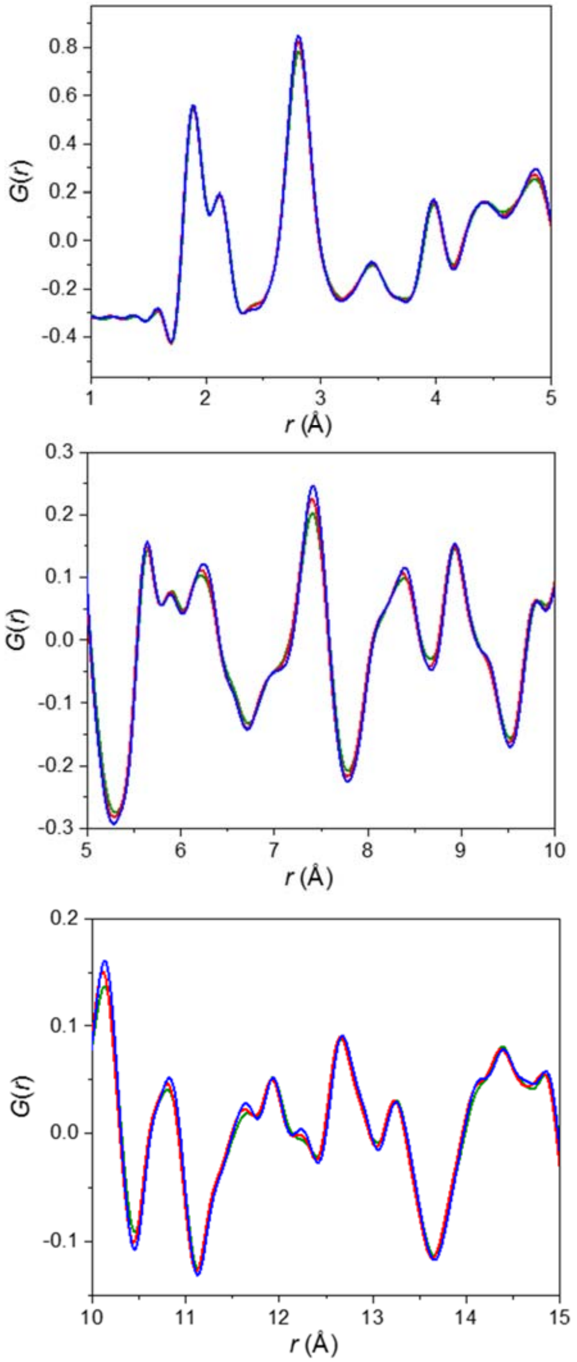

Fig. S3: Comparison of different sections of the experimental neutron pair-distribution functions, $G_N(r)$, for the solid solution samples. Blue – x=0.47, red – x=0.53, green – x=0.58. Small systematic differences are observed for the O-O, A-K/Na peaks. Note that these small differences among the experimental PDFs were closely reproduced by the RMC fits.

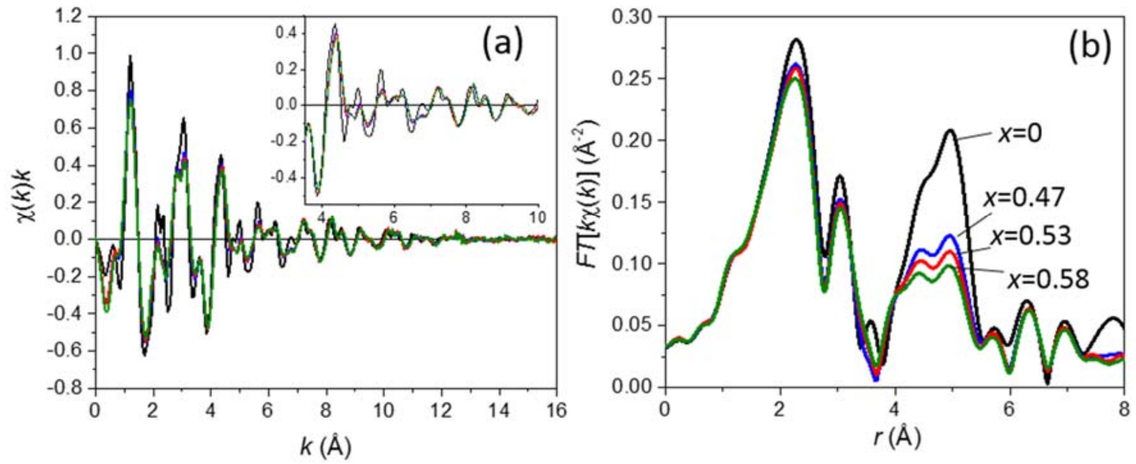

Fig. S4: Comparison of the K EXAFS data for different *x*-values. Black – x=0, Blue – *x*=0.47, red – *x*=0.53, green – *x*=0.58.

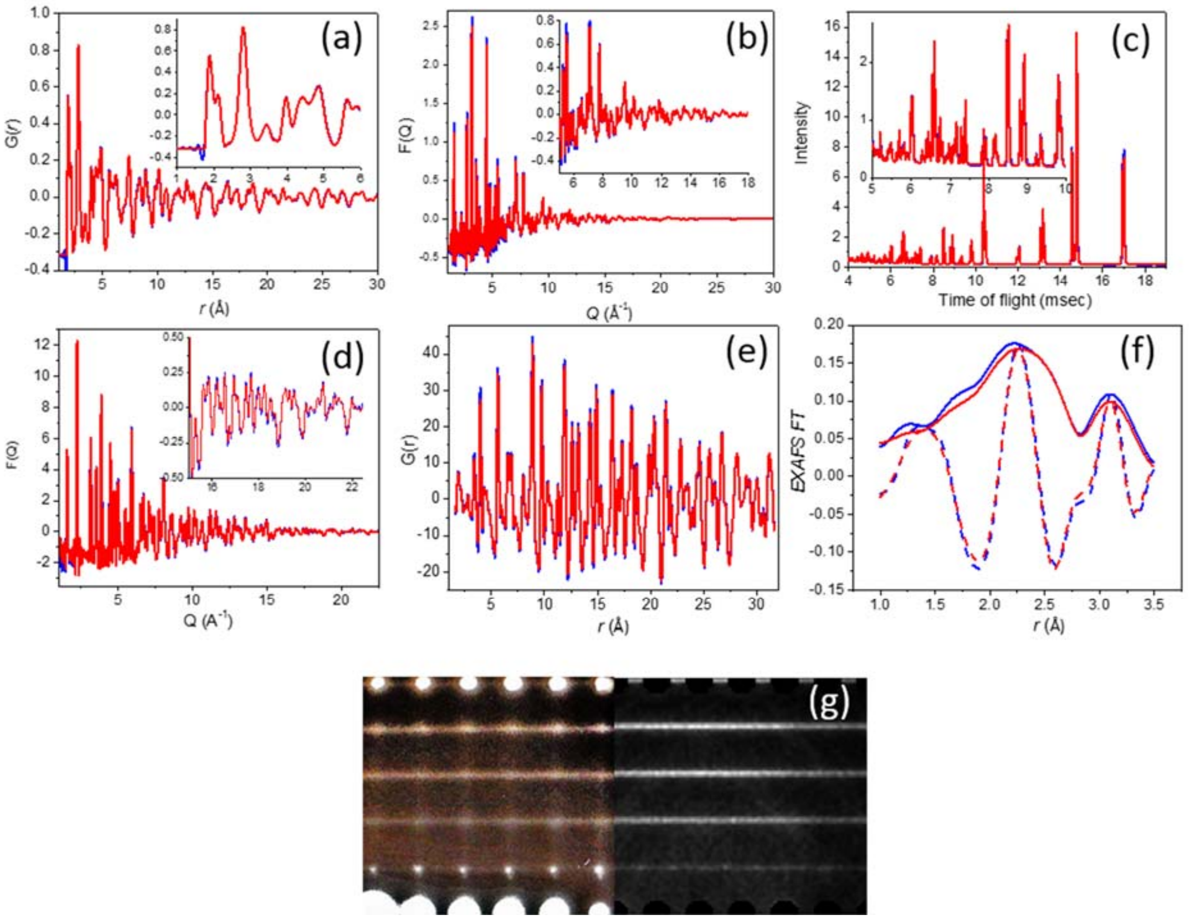

Fig. S5: Experimental and calculated patterns for x=0.53. (a) G_N(r), (b) S_N(Q), (c) neutron Bragg profile, (d) X-ray S_X(Q), (e) X-ray G_X(r), (f) K EXAFS, (g) electron diffuse scattering (the experimental and calculated {150} patterns are shown in the left and right parts of the figure, respectively). For

EXAFS, the real part and the total magnitude of the signal Fourier transform are is shown. The *k* range used in the FT is from ≈2.7 Å^{-1} to ≈10 Å^{-1} and the fitting range in *r*-space is from 1 Å to 3.5 Å. The K-O, K-Nb, and K-K single scattering as well as all the multiple-scattering paths within this *r*-range were included in the fit.
